# Low-dose naltrexone for treatment of pain in patients with fibromyalgia: a randomized, double-blind, placebo-controlled, crossover study

**DOI:** 10.1097/PR9.0000000000001080

**Published:** 2023-06-15

**Authors:** Kirsten Bested, Lotte M. Jensen, Trine Andresen, Grete Tarp, Louise Skovbjerg, Torben S.D. Johansen, Anne V. Schmedes, Ida K. Storgaard, Jonna S. Madsen, Mads U. Werner, Anette Bendiksen

**Affiliations:** a Multidisciplinary Pain Clinic, Friklinikken, Grindsted, Denmark; b Molecular Diagnostics and Clinical Research Unit, Hospital Sonderjylland, Aabendraa, Denmark; c Multidisciplinary Pain Center, Neuroscience Center, Rigshospitalet, Copenhagen University Hospitals, Copenhagen, Denmark; d Department of Economics, University of Southern Denmark, Odense, Denmark; e Department of Clinical Biochemistry and Immunology, Lillebaelt Hospital, University Hospital of Southern Denmark, Vejle, Denmark; f Department of Drug Design and Pharmacology, Copenhagen University Hospitals, Copenhagen, Denmark; g Department of Regional Health Research, University of Southern Denmark, Odense, Denmark

**Keywords:** Low-dose naltrexone, Fibromyalgia patients, Chronic pain, RCT

## Abstract

**Introduction:**

Fibromyalgia (FM) is a chronic fluctuating, nociplastic pain condition. Naltrexone is a µ-opioid-receptor antagonist; preliminary studies have indicated a pain-relieving effect of low-dose naltrexone (LDN) in patients with FM. The impetus for studying LDN is the assumption of analgesic efficacy and thus reduction of adverse effects seen from conventional pharmacotherapy.

**Objectives:**

*First*, to examine if LDN is associated with analgesic efficacy compared with control in the treatment of patients with FM. *Second*, to ascertain the analgesic efficacy of LDN in an experimental pain model in patients with FM evaluating the competence of the descending inhibitory pathways compared with controls. *Third,* to examine the pharmacokinetics of LDN.

**Methods:**

The study used a randomized, double-blind, placebo-controlled, crossover design and had a 3-phase setup. The first phase included baseline assessment and a treatment period (days −3 to 21), the second phase a washout period (days 22–32), and the third phase a baseline assessment followed by a treatment period (days 33–56). Treatment was with either LDN 4.5 mg or an inactive placebo given orally once daily. The primary outcomes were Fibromyalgia Impact Questionnaire revised (FIQR) scores and summed pain intensity ratings (SPIR).

**Results:**

Fifty-eight patients with FM were randomized. The median difference (IQR) for FIQR scores between LDN and placebo treatment was −1.65 (18.55; effect size = 0.15; *P* = 0.3). The median difference for SPIR scores was −0.33 (6.33; effect size = 0.13; *P* = 0.4).

**Conclusion:**

Outcome data did not indicate any clinically relevant analgesic efficacy of the LDN treatment in patients with FM.

## Abstract 2

Supplemental Digital Content is Available in the Text.

## 1. Introduction

Low-dose naltrexone (LDN), in daily doses of 1 to 5 mg,^45^ is, due to its potential analgesic and anti-inflammatory effects, increasingly used as an off-label treatment of fibromyalgia (FM) and some autoimmune pain conditions.^10,36,37,45,57^ Naltrexone is well-known for its use in the treatment of opioid and alcohol addiction in daily doses of at least 50 mg. Naltrexone is a µ-opioid-receptor antagonist with, to a lesser extent, δ-receptor antagonistic properties, bearing a close structural similarity to naloxone. Increased oral bioavailability and longer half-life (T_1/2β_) for the active metabolite 6β-naltrexol makes naltrexone pharmacologically preferable over naloxone.^57^ Recent experimental studies have demonstrated that LDN acts as an immune modulator in the CNS.^19,31^ Low-dose naltrexone has been used in disorders with a putative significant neuroinflammatory component, eg, chronic pelvic pain, CRPS (complex regional pain syndrome), interstitial cystitis, epilepsy, FM, inflammatory bowel disease, and multiple sclerosis.^24,32,57^

Fibromyalgia is a chronic, nociplastic,^2,4^ musculoskeletal disease with an unknown etiology characterized by widespread fluctuating pain, fatigue, low quality of sleep, and high incidences of depression and anxiety disorders.^23,43^ In Europe and the United States, 2% to 8% of the population experience FM,^12^ affecting women more frequently than men.^7,55^ The main drug classes recommended in FM are antidepressants, anticonvulsants, and opioids.^2,30^ Although demonstrating clear evidence for analgesic efficacy, these drugs are associated with a risk of initiating severe arrhythmias, cardiac dysfunction, neuropsychiatric disorders, serotonergic syndrome, and enhanced postoperative morbidity.^18^ Anticonvulsants have been recommended as alternatives associated with the development of dependence, substance abuse, and suicidality.^27,33^ European League Against Rheumatism (EULAR) recommends that tramadol can be used in severe pain when nonpharmacological multimodal therapies fail.^25^ Paradoxically, in clinical FM studies, opioids generally demonstrate limited analgesic efficacy,^15^ but because tramadol also has serotonin-norepinephrine reuptake Inhibitor (SNRI) properties, this may explain the observed weak analgesic effect. All opioids, including tramadol,^40^ carry a risk for the development of tolerance, dependence, and substance abuse, highlighted by the “opioid epidemic” in the United States.^30,47^

Small-scale studies^10,55,56^ indicate analgesic efficacy of LDN with few adverse effects, and thus, our rationale for this study was to corroborate the findings in a higher volume.

Only 6 studies address LDN as a treatment of FM. For example, a pilot study by Younger et al. described lowered symptoms in all included 10 patients with FM when receiving LDN.^55^ A randomized, double-blind, placebo-controlled study also by Younger et al.^56^ showed a significant decrease in pain intensity in 31 included patients with FM. A study investigating the dose–response relationship among 25 patients with FM^10^ found that 4.5 mg/day was effective in 95%. A study including 8 patients with FM showed a decrease in proinflammatory cytokines and less pain and symptoms after 8 weeks of LDN treatment.^31^ An explorative study describing cold pressor test (CPT) to measure pain included 15 patients with FM and showed improved scores after receiving LDN.^29^ Finally, an explorative study using CPT in patients with chronic opioid treatment showed improved CPT scores among 21 patients with FM after LDN treatment.^22^

The main objectives of this study were as follows: *First*, to examine if LDN was associated with a higher analgesic efficacy and improvement in physical function score compared with placebo. *Second*, to ascertain the analgesic efficacy of LDN in experimental pain procedures using quantitative somatosensory testing.^9,28^ *Third,* to examine the pharmacokinetics of LDN and the main metabolite 6β-*naltrexol*.

## 2. Methods

### 2.1. Study management

The Committee of Health Research Ethics of The Region of Southern Denmark (S-20150159), the Danish Medicines Agency (2015102044), and the Data Inspection Authority of The Region of Southern Denmark (2008-58-0035) approved the study protocol. The study was registered in EUDRACT (2015-002972-26) and ClinicalTrials.gov (NCT02806440) and conducted in accordance with Good Clinical Practice (GCP) and Good Manufacturing Practice (GMP).

### 2.2. Investigational centers

The study was planned as a 2-center collaboration between The Multidisciplinary Pain Center at Rigshospitalet, Copenhagen (MPC-C), a tertiary university facility, and The Multidisciplinary Pain Clinic at Friklinikken, Grindsted, (MPC-G), a secondary health care facility. In total, an enrollment of 140 patients with FM was planned, with 70 patients with FM allocated at each investigational center. Due to organizational changes at MPC-C, only 1 patient was randomized at MPC-C. This patient made a withdrawal of consent after intake of 1 tablet. The study continued as 1 investigational center, MPC-G, planned to include 70 patients with FM.

### 2.3. Study design

#### 2.3.1. Study setting

**Figure 1.**
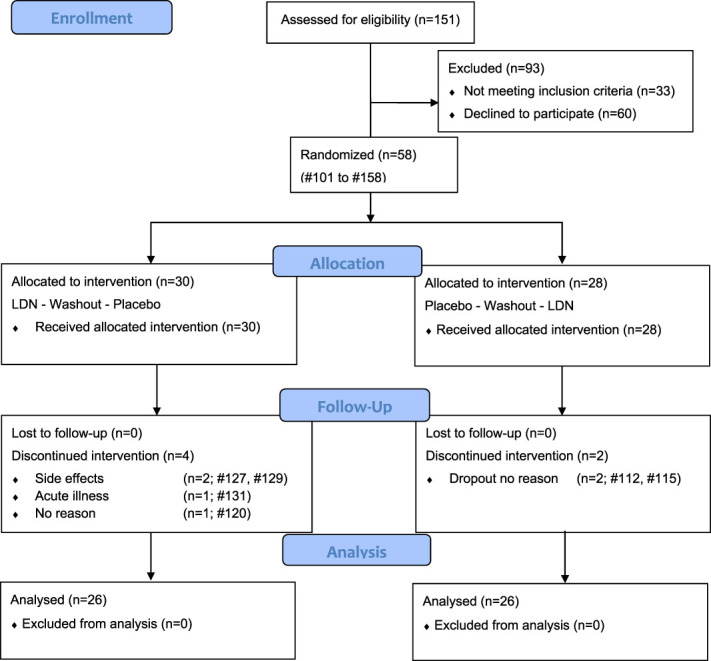
Consort (2010) flow diagram, patient selection, and eligibility. LDN, low-dose naltrexone.

The study was an investigator-initiated and investigator-driven study using a block-randomized, double-blind, placebo-controlled, crossover design (see Fig. 1 showing overview of study). The patients with FM were randomized to treatment with LDN or placebo in the first or second treatment period. The patients with FM were randomized and allocated equally according to computer random table method. The pharmacy managed the randomization sequence generated by a web-based randomization site.^4^ The sequence was generated using the second generator function, applying blocks of 10 and balanced permutations (see text, Supplemental Digital Content 1, text describing study setting, available at http://links.lww.com/PR9/A195).

The randomization lists were stored in secure and locked confines, only accessible by the principal investigator. In case of a medical emergency, the code could be individually unblinded. Patients with FM were included consecutively and evenly over time. New patients with new randomization numbers were allocated to replace dropouts. Patients, study staff, and staff in pain clinic were blinded throughout the study period. The study was executed transparently and presented in accordance with CONSORT guidelines. Information and data collection from patients were done by 5 trained staff members. Study data were manually entered into an electronic case report form (e-CRF). After the last patient visit, data were stored in OPEN, a dedicated research registry in The Region of Southern Denmark (see text, Supplemental Digital Content 1, text describing study setting and source data, available at http://links.lww.com/PR9/A195).

### 2.4. Outcomes

#### 2.4.1. Primary outcomes

The primary outcomes were patients with FM reporting on function, total impact, and symptoms in Fibromyalgia Impact Questionnaire revised (FIQR)^5^ questionnaire (cf. 2.8.2.1) and reporting on pain intensity using the summed pain intensity ratings (SPIR)^8,26^ (cf. 2.8.2.2).

#### 2.4.2. Secondary outcomes

The secondary outcomes were as follows:

(1) Diary-based questionnaires; Brief Pain Inventory-Short Form (BPI-SF),^51^ Daily Sleep Interference Scale (DSIS),^48^ Hospital Anxiety and Depression Scale (HADS),^58^ PainDETECT Questionnaire (PD-Q),^16^ and Pain Catastrophizing Scale (PCS)^35,42^ (cf. 2.8.2.3)
(2) Quantitative Somatosensory Testing (QST)^28^ paradigms (cf. 2.9)
(3) Pharmacokinetics of naltrexone and the main metabolite 6β-naltrexol (cf. 2.10.2)

#### 2.4.3. Timeline

**Figure 2.**
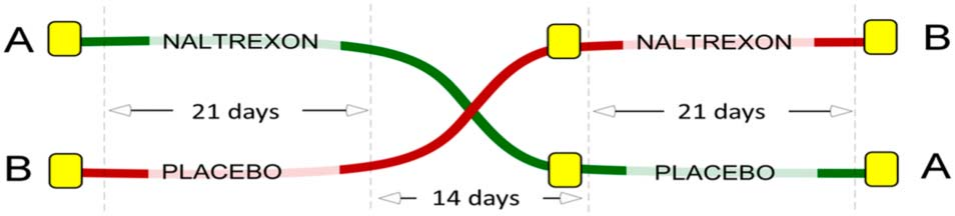
Study design. Patients were randomized and allocated in a double-blind design to follow (A) or (B). The 2 treatment periods were identical (21 days) and separated by a washout period (14 ± 2 days). Before each treatment period, baseline assessments (questionnaires, experimental pain testing, and blood samples) were performed, providing a clinical status of the patient.

The study had a 3-phase setup (see Fig. 2 showing study phases). The first phase included baseline assessment (BA1) (day −3 to day 1) and a treatment period (day 1 to day 21) including an outcome assessment (OA1). The second phase was a washout period (day 22 to day 32). The third phase included a baseline assessment (BA2) (day 33 to day 36) followed by a treatment period (day 36 to day 56) including an outcome assessment (OA2). The patients with FM attended 6 separate examination days (see Table 1, showing overview of the study).

**Table 1:** Overview of the study showing the 3 phases including 2 treatment periods separated by the washout period.

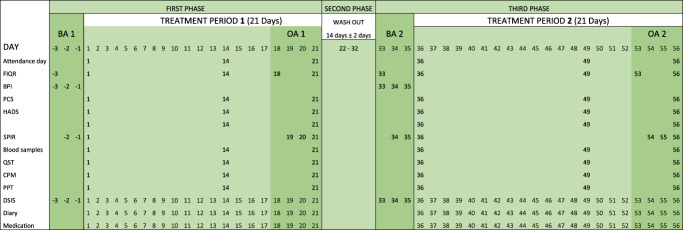

The first and second baseline assessment (BA1/BA2) and the first and second outcome assessment (OA 1/OA 2) are shown. Detailed day-by-day timetable for the completion of examination days, study questionnaires, pain ratings, blood sampling, quantitative sensory testing, diary, and medication are shown.

BA1, baseline assessment period 1; BA2, baseline assessment period 2; BPI, Brief Pain Inventory - Short Form; CPM, conditioned pain modulation; Diary, days for completion the diary; DSIS, Daily Sleep Interference Scale; FIQR, Fibromyalgia Impact Questionnaire revised; HADS, Hospital Anxiety and Depression Scale; Medication, days with consumption of project medication LDN/Placebo; OA1, outcome assessments period 1; OA2, outcome assessments period 2; PCS, pain catastrophizing scale; PPT, pain pressure threshold; PD-Q, PainDetect Questionnaire; QST Hyperalgesia + Allodynia, heat/capsaicin test; SPIR, summed pain intensity rating.

### 2.5. Drugs

Naltrexone (4.5 mg) and identical placebo tablets were manufactured and packed in a blinded and randomized fashion by the pharmacy (magistral production, Glostrup Apotek, Copenhagen, Denmark). Naltrexone (4.5 mg) is not marketed in Denmark but manufactured by permission of the Danish Medicines Agency (DMA), controlling the authorization and licensing of the manufacturing process according to GMP. Naltrexone 4.5 mg was chosen for this study because it is the typically applied dosage in existing studies.^39,56^

### 2.6. Patients

All patients with FM were screened by a medical specialist in rheumatology and fulfilled the ACR's (American College of Rheumatology) 2011 criteria of FM^3,52–54^ before enrollment in the study. The patients with FM were recruited from the patient registries at MPC-G.

#### 2.6.1. Inclusion and exclusion criteria

Inclusion and Exclusion criteria are indicated in Table 2 (see Table 2, showing inclusion and exclusion criteria).

**Table 2:** Inclusion and exclusion criteria.

Inclusion criteria
≥18 y of age
Diagnosed with fibromyalgia according to the criteria of ACR* by a rheumatologist
Premenopausal women in contraceptive treatment† or sterilized
Patient referred to multidisciplinary pain treatment
Exclusion criteria
Other inflammatory rheumatologic diseases
Pregnant/lactating
Opioid treatment
Cancer diagnosis
Unstable analgesic medication‡
Non-proficient in Danish or English
Allergy towards opioids
Severe hepatic insufficiency
Severe renal insufficiency
Acute pancreatitis
Patients were withdrawn from the study if the investigator/sub-investigator deemed it necessary due to medical reasons.

* American College of Rheumatology (2011 Wolfe F, Häuser W. Fibromyalgia diagnosis and diagnostic criteria. Ann Med 2011; 43:495–502).

† Contraceptives defined as spiral or hormonal contraceptive drugs (birth control tablets, implants, transdermal depot patches, vaginal ring, or injectable depot).

‡ Paracetamol rescue is accepted (requires registration in the diary).

### 2.7. Concomitant treatment

Concomitant medications were registered in the e-CRF, including generic names and doses. Paracetamol was used as rescue medication (1 g maximum 3 times a day).

### 2.8. Study chronology

#### 2.8.1. Diary

The patients with FM received a diary that allowed entries concerning study medication, adverse events, pain assessment forms, questionnaires, and adverse events.

#### 2.8.2. Questionnaires

The questionnaires were self-reported. Patients were contacted by phone the day before answering the first questionnaire.

##### 2.8.2.1. Fibromyalgia impact questionnaire revised

The FIQR^5^ is a tool developed to assess FM-related problems and response to a given treatment. The FIQR was translated from the original English version to Danish by 4 health care professionals specialized in the management of chronic pain. The back-translation was then performed by a native English individual fluent in Danish. After revision and back-translation, a final revised Danish version of FIQR was generated. The FIQR explores 3 domains: function, total impact, and symptoms. The patient was asked to answer based on the experience during the last 7 days before filling in the questionnaire. The questionnaire includes 21 questions regarding everyday activities. The patient was asked to mark the degree of difficulty spanning from “no difficulty” to “very difficult performing the activity.” Furthermore, the questionnaire evaluated whether the patient was restricted or incapacitated in doing the weekly chores by the FM symptoms. The FIQR also assessed current pain intensity, energy level, sleep quality, anxiety symptoms, feeling depressed, body stiffness, sensitivity to touch, difficulties with balance, memory, and with the perception of loud, shrill noises, smells, or cold.

The FIQR scoring was made by dividing the function domain sum (0–90 points) by 3. The overall impact domain was left unchanged (0–20 points). The symptom domain sum (0–100 points) was divided by 2. The 3 domain scores were then summed (0–100 points). Differences in mean score in FIQR between BA1 (day −3 and day 1) and BA2 (day 33 and day 36) and between OA1 (day 18 and day 21) and OA2 (day 53 and day 56), respectively, were calculated.

##### 2.8.2.2. Numeric rating scale, summed pain intensity rating

The 11-point numeric rating scale (NRS)^8,26^ was applied to evaluate pain intensity (during rest, personal hygiene measures, and activity of daily living). The patient with FM indicated the pain intensity (0–10; 0 = “no pain”; 10 = “worst possible pain”) based on the experience during the last 24 hours before filling in the questionnaire. The NRS ratings across each activity were summed as SPIR (0–30 points). Differences in mean score in SPIR between BA1 (days −2, −1, 1) and BA2 (days 34, 35, 36) and between OA1 (days 19, 20, 21) and OA2 (days 54, 55, 56), respectively, were calculated.

##### 2.8.2.3. Miscellaneous questionnaires

The BPI-SF,^51^ DSIS,^48^ HADS,^58^ PD-Q,^16^ and PCS^42^ are described in detail in Supplemental Digital Content (see text, Supplemental Digital Content 2, text describing miscellaneous questionnaires, available at http://links.lww.com/PR9/A195).

### 2.9. Quantitative somatosensory testing

Quantitative somatosensory testing (QST) is a standardized activation of the sensory system by the application of graded chemical, electrical, mechanical, or thermal test stimuli, with an assessment of the evoked psychophysical responses, examining sensory detection and pain thresholds.^28^

#### 2.9.1. Heat–capsaicin sensitization

The heat–capsaicin sensitization test is a validated experimental pain model investigating aspects of central sensitization, eg, secondary hyperalgesia and allodynia^13,34,38^ (see text, Supplemental Digital Content 3, text describing quantitative somatosensory testing, available at http://links.lww.com/PR9/A195).

#### 2.9.2. Pressure pain thresholds

Assessments of pressure pain thresholds (PPTs) were performed with a calibrated pressure algometer^34^ (see text, Supplemental Digital Content 3, text describing quantitative somatosensory testing, available at http://links.lww.com/PR9/A195).

#### 2.9.3. Conditioned pain modulation test

The conditioned pain modulation (CPM) test evaluates the efficiency of the descending inhibitory pathways and has been used as a quantitative measure of pain disinhibition in patients with FM.^9^ The CPM test was performed as a cold pressor test; PPT1 was the baseline assessment, and PPT2 the assessment measured after the patient had submerged their left hand in cold water for 60 seconds (see text, Supplemental Digital Content 3, text describing quantitative somatosensory testing, available at http://links.lww.com/PR9/A195).

The CPM efficiency was calculated as follows:

$$\text{CPM efficiency }\left(\text{\%}\right)=\frac{100\text{\%}\times \left(PPT2-PPT1\right)}{PPT1}.$$

### 2.10. Blood Sampling

#### 2.10.1. Routine blood chemistry

Screening of kidney and liver function was tested before inclusion in the study according to safety criteria.

#### 2.10.2. Naltrexone and 6β-naltrexol plasma concentration measurements

To examine the pharmacokinetics (PK) of naltrexone and its main metabolite, 6β-naltrexol, venous blood samples were collected in lithium-heparin–containing tubes on days 1, 14, 21, 36, 49, and 56 (see text, Supplemental Digital Content 4, text describing blood sampling and analysis, available at http://links.lww.com/PR9/A195).

On days 1 and 36 (first day of treatment periods), samples were collected in the morning just before the intake of the first tablet and then subsequently at 15, 30, 45, and 60 minutes after tablet intake. On days 14, 21, 49, and 56, medication was taken in the morning, and samples were collected during the clinical visit 1 to 3 hours later.

### 2.11. Adverse events

Definitions, monitoring, and reporting procedures are described in Supplemental Digital Content 5 (see text, Supplemental Digital Content 5, text describing adverse events, available at http://links.lww.com/PR9/A195).

#### 2.11.1. Safety

Low-dose naltrexone is considered safe to administer. Four studies^19,55–57^ only reported mild adverse events. The following adverse events were specifically asked for and reported: sleep disturbances, vivid dreams, nausea, diarrhea, headache, and tiredness.

### 2.12. Statistics

#### 2.12.1. Statistical significance

The authors are aware of the discussions concerning the indiscriminate use of *P* values as an absolute mean of null hypothesis testing.^1,49,50^ In this article, the term statistical significance was avoided. The advice “correct and careful interpretation of statistical tests demands examining the sizes of effect estimates and confidence limits, as well as precise *P* values (not just whether *P* values are above or below 0.05 or some other threshold)” was generally followed.^6,17,20^

#### 2.12.2. Sample size estimates

The calculation is based on FIQ data from Younger et al.,^56^ where mean values (SD) in the LDN group of 28.8 (12.5)% and in the placebo group of 18.0 (14.6)% are given for pain reduction, which gives an effect size (ES) of 0.61 (GPower*3.1.9.2, Kiel University, Germany).

The sample size estimates were based on a 1% chance of type Ⅰ errors (α = 0.01), 10% chance of type Ⅱ errors (β = 0.10), nonparametric distribution (ARE-correction; paired analysis with Wilcoxon signed-rank test), and an estimated correlation coefficient (*r*) between the treatments of 0.3. The estimated sample size per center was 51, allowing complete analyses to be performed at each center (see text, Supplemental Digital Content 6, text describing statistics, available at http://links.lww.com/PR9/A195).

#### 2.12.3. Statistical data processing

Our analyses focused on measuring the pharmacodynamics effects of LDN compared with placebo on a number of primary and secondary outcomes. We exploited our access to both baseline and outcome measures for all individuals under both active treatment and placebo. This allowed us to perform paired tests. First, baseline (BA) and outcome (OA) measures were transformed into measures of changes (Δ) for all variables (*v*) and for all individuals (*i*) under treatment with LDN or placebo:

$$\Delta v_{i}=v_{i}^{OA}-v_{i}^{BA}.$$

To assess the pharmacodynamics effects of LDN, the differences between LDN treatment and placebo were examined (see text, Supplemental Digital Content 6, text describing statistics, available at http://links.lww.com/PR9/A195). Formally testing the null hypotheses of no effects, paired Wilcoxon signed-rank tests were used to report the associated *P* values and appropriate ES. For completeness, the results of the corresponding parametric tests were reported in tabular form (mean [SD], *P*, 95% confidence interval [CI], effect sizes [ESs]). Data are reported as median (IQR) unless otherwise stated.

## 3. Results

### 3.1. Patients

A total of 151 patients with FM were assessed for eligibility, and 58 patients with FM were included and randomized. Sixty patients declined to participate mostly due to time requirement, and 33 did not meet inclusion criteria. Two patients with FM dropped out due to adverse events (nausea, vomiting) immediately after the treatment started. One patient dropped out due to concomitant acute illness, and 3 patients with FM did not state the withdrawal reason. Fifty-two patients with FM fulfilled the study per protocol. The first patient was included in May 2016, and the last patient visit was in December 2019. Inclusion was evenly distributed over time.

#### 3.1.1. Concomitant medication and demographics

Demographic data and use of concomitant medication are described in Table 3 (see Table 3 describing demographic) and Table 4 (see Table 4 describing concomitant medication), respectively. The patients with FM continued the medication in stable doses during the study.

**Table 3:** Demographics data.

Demographics	Value	Mean
Male/female, n	6/46	—
Age min/max, y	24/66	50.4
BMI min/max, kg/m^2^	20/41	31.2
Weight min/max, kg	52/130	86.5

BMI, body mass index.

**Table 4:** Concomitant medication.

Concomitant medication	n*	DD† mg
Paracetamol‡	31	500
Paracetamol§	8	1000–4000
Tizanidine	12	2–10
Baclofen	2	10–20
Chlorzoxazone	2	375–1500
Gabapentin	2	60–800
Pregabalin	6	150–300
Lamotrigine	1	200
Topiramate	1	200
Amitriptyline	5	10–90
Duloxetine	5	60–120
Venlafaxine	4	75–300
Citalopram	2	20–40
Sertraline	1	100
Levodopa	3	125
Melatonin	3	3
Eletriptan‡	1	40
Sumatriptan‡	1	100
Metformin	1	1000

* Number of patients with intake.

† Daily dose.

‡ Prn.

§ Fixed daily dose.

#### 3.1.2. Adverse events

Adverse events (see text, Supplemental Digital Content 5, text describing adverse events, available at http://links.lww.com/PR9/A195) were registered at visit days in both treatment periods. Headache, fatigue, nausea, and dizziness were registered in both treatment periods by a small number of patients with FM, and all adverse events were classified as minor (See Table 5 describing adverse events). The 2 patients with FM who dropped out immediately after the start of the treatment experienced minor adverse events.

**Table 5:** Patients reported adverse events.

Adverse event	LDN treatment (n)	Placebo treatment (n)
Headache	9	9
Fatigue	9	4
Nausea	8	5
Dizziness	7	4
Stomach ache	3	4
Diarrhea	3	2
Constipation	2	2
Blurry vision	1	2
Worsened sleep	0	3
Improved sleep	1	3
Pain more intense	2	1

Adverse events reported in dairy by patient during each treatment period (n = 52). All adverse events were classified as minor, and no serious adverse events or suspected unexpected adverse reactions occurred.

LDN, low-dose naltrexone.

### 3.2. Primary outcome

#### 3.2.1. Fibromyalgia impact questionnaire revised

**Figure 3.**
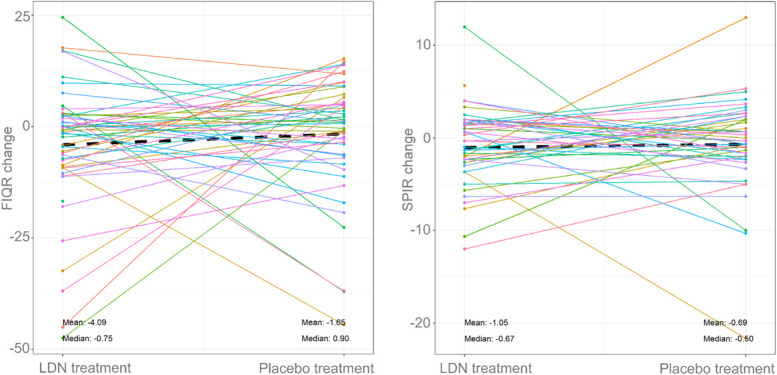
Dot-line diagram presenting change in FIQR score (left panel) and SPIR score (right panel) after LDN treatment and placebo treatment. Each line represents a patient, mean and median values for all patients shown in bottom of figure. Negative values (below 0) indicate improvements. Black dashed line indicates mean value at LDN and placebo treatment. FIQR, Fibromyalgia Impact Questionnaire revised; LDN, low-dose naltrexone; SPIR, summed pain intensity ratings.

Baseline and outcome scores for FIQR were obtained under both LDN and placebo treatment (n = 50). The differences were −1.65 (18.55) (see Fig. 3 dot-line diagram showing scores). The Wilcoxon signed-rank test did not indicate any difference between LDN and placebo (ES = 0.15, CI = −6.72 to 2.15; *P* = 0.30; see Table 6 describing FIQR results). The random effects model did not indicate any difference between LDN and placebo (conditional mean difference −2.50, *P* = 0.34) (see text, Supplemental Digital Content 6, text describing statistical data processing, available at http://links.lww.com/PR9/A195; see Table 7 describing absolute measures).

**Table 6:** Changes in primary outcome variables from low-dose naltrexone treatment to placebo treatment.

Outcome	n	Median	IQR	*P*	CI (95%)	ES	Mean	SD	*P*	CI (95%)	Cohen d (ES)
FIQR (score)	50	−1.65	18.55	0.30	−6.72, 2.15	0.15	−2.19	20.40	0.45	−7.98, 3.61	−0.11
SPIR (score)	45	−0.33	6.33	0.40	−2.17, 0.92	0.13	−0.23	6.48	0.24	−2.18, 1.72	−0.04

ES, effect size; FIQR, Fibromyalgia Impact Questionnaire revised; IQR, interquartile range; n, number of patients; SPIR, summed pain intensity rating.

**Table 7:** Changes in primary outcome variables.

Outcome	BA1 n = 52		OA1 n = 52		Change from BA1 to OA1		BA2 n = 51		OA2 n = 51		Change from BA2 to OA2	
FIQR score (0–100)	Mean	SD	Mean	SD	Mean	SD	Mean	SD	Mean	SD	Mean	SD
LDN treatment	53.8	2.4	48.7	3.5	−5.0	2.8	49.8	3.7	46.7	3.7	−3.0	2.7
Placebo treatment	52.1	3.7	46.5	3.9	−5.6	3.0	53.8	3.3	56.3	3.3	2.5	1.5

Outcome	BA1 n = 48		OA1 n = 48		Change from BA1 to OA1		BA2 n = 44		OA2 n = 44		Change from BA2 to OA2	
SPIR score (0–30)	Mean	SD	Mean	SD	Mean	SD	Mean	SD	Mean	SD	Mean	SD
LDN treatment	16.6	0.9	15.4	1.2	−1.2	0.9	16.2	1.0	15.4	1.0	−0.8	0.8
Placebo treatment	17.1	1.0	15.3	1.4	−1.7	1.1	17.6	1.1	18.1	1.2	0.4	0.8

FIQR, Fibromyalgia Impact Questionnaire revised; SPIR, summed pain intensity rating, absolute values.

Scores of FIQR respectively SPIR at BA1, baseline assessment 1; OA1, outcome assessment 1; BA2, baseline assessment 2; OA2, outcome assessment 2; n, number of patients.

#### 3.2.2. Summed pain intensity ratings

The difference in SPIR scores between LDN and placebo was −0.33 (6.33) (see Fig. 3 dot-line diagram showing scores). The Wilcoxon signed-rank test revealed ES of 0.13 and CI of −2.17 to 0.92 (*P* = 0.4; see Table 6 describing SPIR results). The random effects model did not indicate any difference between LDN and placebo (conditional mean difference −0.40, *P* = 0.68) (see text, Supplemental Digital Content 6, text describing statistical data processing, available at http://links.lww.com/PR9/A195; see Table 7 describing absolute measures).

### 3.3. Secondary outcomes

See Table 8 describing secondary outcomes results.

**Table 8:** Changes in secondary outcome variables from low-dose naltrexone treatment to placebo treatment.

Outcome	n*	Median	IQR	*P*	CI (95%)	ES	Mean	SD	*P*	CI (95%)	Cohen d (ES)
Hyperalgesia (cm^2^)	33	−0.9	48.1	0.83	−14.6, 12.1	0.04	0.0	37.2	1.00	−13.2, 13.2	0.00
Allodynia (cm^2^)	33	−14.5	36.5	0.17	−20.8, 5.	0.24	−6.7	36.5	0.30	−19.6, 6.3	−0.18
PPT tender (kPa)	32	0.1	0.5	0.88	−0.2, 0.2	0.03	−0.0	0.8	0.84	−0.3, 0.3	−0.04
PPT control (kPa)	34	0.1	0.8	0.75	−0.3, 0.3	0.06	0.0	0.9	0.98	−0.3, 0.3	0.00
CPM efficiency (%)	34	−9.3	61.0	0.23	−25.4, 6.9	0.21	−9.3	46.3	0.40	−25.4, 6.9	−0.14
HADS A (score)	48	−1.0	3.3	0.06	−2.0, 0.0	0.28	−1.0	3.3	0.04	−2.0, 0.1	−0.31
HADS D (score)	48	1.0	3.3	0.22	−0.5, 2.0	0.22	0.5	3.5	0.32	−0.5, 1.5	0.14
PCS R (score)	33	0.0	8.0	0.65	−3.0, 1.0	0.11	−0.2	4.7	0.84	−1.9, 1.5	−0.04
PCS M (score)	32	−0.5	4.0	0.10	−2.5, 0.0	0.27	−0.7	2.9	0.17	−1.8, 0.3	−0.25
PCS H (score)	34	−0.5	8.5	0.48	−3.5, 1.5	0.12	−1.1	6.6	0.34	−3.4, 1.2	−0.16
DSIS (score)	38	0.2	2.5	0.75	−0.6, 1.0	0.07	0.3	2.5	0.45	−0.5, 1.2	0.12

* Technical problems with the pressure algometer and the thermal analyzer reduced the available number of quantitative somatosensory assessments.

CPM, conditioned pain modulation; DSIS, Daily Sleep Interference Scale; ES, effect size; HADS, Hospital Anxiety and Depression Scale (A = Anxiety, D = Depression); IQR, interquartile range; n, number of patients; PPT Control, pressure pain threshold at control points; PCS, pain catastrophizing scale (R = rumination, M = magnification, H = helplessness); PPT, pressure pain threshold at tender points.

#### 3.3.1. Quantitative somatosensory testing

##### 3.3.1.1. Secondary hyperalgesia areas

The differences in secondary hyperalgesia areas between LDN and placebo treatments (n = 33) were −0.88 (48.13) cm^2^. Wilcoxon signed-rank test revealed ES of 0.04 and CI of −14.63 to 12.10 (*P* = 0.83).

##### 3.3.1.2. Allodynia

The differences in allodynia areas between LDN and placebo treatments (n = 33) were −14.46 (42.04) cm^2^. Wilcoxon signed-rank test revealed ES of 0.24 and CI of −20.82 to 4.96 (*P* = 0.65).

##### 3.3.1.3. Pressure pain threshold

The differences in PPT (kPa) at tender points between LDN and placebo treatments (n = 32) were 0.08 (0.52) kPa. Wilcoxon signed-rank test revealed ES of 0.03 and CI and −0.21 to 0.21; *P* = 0.88. The differences in PPT at control points between LDN and placebo treatments (n = 34) were 0.08 (0.76) kPa. Wilcoxon signed-rank test revealed ES of 0.06 and CI −0.26 to 0.29 (*P* = 0.75).

##### 3.3.1.4. Conditioned pain modulation

The difference in CPM (%) between LDN and placebo was (n = 34) −9.25 (61.00). Wilcoxon signed-rank test revealed ES of 0.21 and CI of −25.42 to 6.93 (*P =* 0.23).

#### 3.3.2. PainDETECT

The PD-Q questionnaire evaluates the likelihood of presence of a neuropathic pain component. At day 1, before treatment, 6 patients with FM scored the neuropathic component to be very unlikely, 22 that the component could not be rejected and 24 that the neuropathic was very likely.

#### 3.3.3. Miscellaneous questionnaires

Differences in scores between LDN and placebo treatment from the questionnaires for HADS, PCS, and DSIS are presented in Table 8 (see Table 8 describing scores in miscellaneous questionnaires).

### 3.4. Blood Samples

#### 3.4.1. Pharmacokinetics and pharmacodynamics

**Figure 4.**
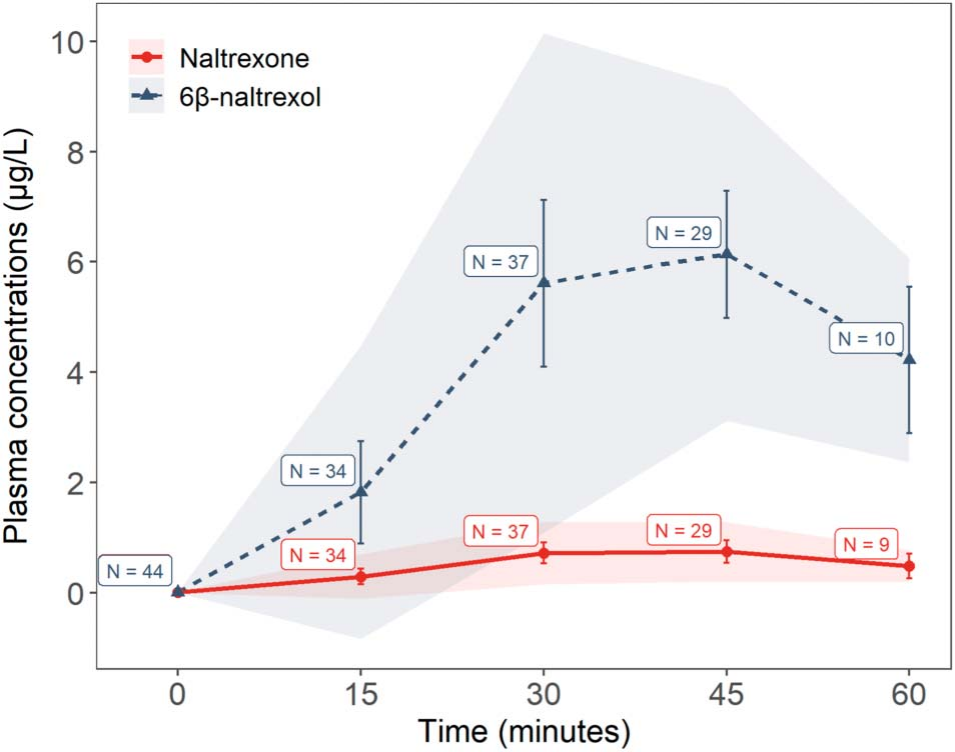
Diagram presenting mean plasma concentrations of naltrexone and 6β-naltrexol after oral intake of 4.5 mg naltrexone at times 0, 15, 30, 45, and 60 minutes. Error bars represent 95% confidence intervals, and shaded areas represent standard deviations. Mean plasma concentrations at 30 minutes were 0.66 µg/L for naltrexone and 4.61 µg/L for 6β-naltrexol.

Plasma concentrations (Cp) of naltrexone and 6β-naltrexol on the first treatment day showed a fast absorption rate of naltrexone and a rapid conversion to 6β-naltrexol for all patients with FM. The more than 10-fold increase in Cp of 6β-naltrexol, compared with naltrexone, is due to first-pass metabolism^11,41^ with a high hepatic extraction ratio for the parent compound. Peak Cp were likely reached at 30 to 45 minutes after ingestion (see Fig. 4 showing plasma concentrations). After 21 days of treatment, samples were taken 1 to 3 hours after tablet intake. The median Cp of naltrexone and 6β-naltrexol were 0.33 µg/L and 5.29 µg/L, respectively. Detailed pharmacokinetic analyses were not performed due to short sampling period.

## 4. Discussion

### 4.1. Outcome

In this randomized controlled study, using a crossover design, 52 patients with FM fulfilling the ACR's 2011 criteria, the efficacy of treatment with LDN, were examined. The outcome data did not indicate any analgesic efficacy or improvement in physical function score related to the treatment. Using experimental tests of neuroplasticity perturbations, no differences were found related to the treatment. The pharmacokinetic analyses showed a rapid and reliable absorption of naltrexone.

### 4.2. Current management strategies

It is generally agreed that management of FM requires a biopsychosocial approach, eg, cognitive behavioral therapy, education, mindfulness-based stress reduction, physical therapies, and physical exercise.^7,46^ Also, pharmacotherapy is needed in patients with FM with severe pain as a component in multimodal rehabilitation.^2,25,36^

### 4.3. The rationale of multimodal pharmacotherapy

Multimodal analgesic pharmacotherapy includes the use of a combination of drugs, often with different pharmacological mechanisms of action. The multimodal concept may demonstrate infra- or supraadditivity, obtaining identical or better analgesic effects at lower doses of each drug compared with monotherapy. In acute pain management, the combination of paracetamol and NSAID has improved analgesic efficacy and caused a reduction in opioid requirement.^44^ The evidence for the efficacy of multimodal pharmacotherapy in chronic pain is scarce. However, the study did not examine any potential additive analgesic effect by combining LDN with an antidepressant or anticonvulsant.

### 4.4. Strengths of the study

*First*, compared with the early FM studies^55,56^ on LDN, methodological aspects have been improved in this study. In the 2009 study^55^ (n = 10), a single-blind, nonrandomized, crossover design was used with a fixed treatment sequence but with a dissimilar number of treatment weeks. In the 2013 study^56^ (n = 31), a randomized, double-blind, placebo-controlled, counterbalanced, crossover design was used, however, also with dissimilar treatment periods, ie, 12 weeks with LDN *vis-á-vis* 4 weeks with placebo, and *no* washout period between treatments. Interestingly, neither an a priori nor a *post hoc* sample size estimate was presented in any of the studies.

*Second*, this study uses the validated FIQR as a primary outcome parameter, including summed resting and dynamic ADL pain ratings. These measures are considered improvements compared with the previously mentioned studies, only using unimodal nondynamic pain ratings as a primary outcome. Generally, the outcomes in this study are in agreement with the recommended patient phenotyping measures in chronic pain from IMMPACT (Initiative on Methods, Measurement, and Pain Assessment in Clinical Trials)^14^ regarding psychometrics, sleep function, and fatigue. This study tested both the ascending *excitatory* pain pathways by the heat/capsaicin sensitization test and the descending *inhibitory* pathways by the CPM test.

*Third*, the pharmacokinetics of LDN were analyzed.

*Fourth*, a priori sample size estimates were based on the completion of 51 per-protocol treated patients with FM at 1 center, meaning that failure to complete inclusions in one of the centers would *not* jeopardize meaningful statistical analysis from the companion center.

### 4.5. Weaknesses of the study

*First*, the length of the study period, 60 days, may have impeded patient compliance, affecting the attrition rate and the number of dropouts. However, the original study^56^ had a duration of 22 weeks, with 28 of 31 patients with FM completing the study, so this would probably not be an issue.

*Second*, a placebo effect was anticipated, particularly in the first treatment period. The design of this study, however, does not allow an estimate of the magnitude of the placebo effect.

*Third,* although patients with FM fulfilled the ACR's 2011 criteria, analysis of the neuroinflammatory component could have characterized patients with FM further.

*Fourth,* analysis of the neuroinflammatory response to LDN could likely identify subgroups of responders but was beyond the scope of this study.

*Fifth,* in this study, patients with FM were diagnosed by a specialist in rheumatology at least months before enrollment. To our knowledge, no literature has described the relevant start window for LDN treatment compared with the time of diagnosis.

### 4.6. In summary

The recommended pharmacological management of FM, antidepressants, anticonvulsants, and opioids^2,25^ are associated with a substantial risk of development of serious adverse effects. Low-dose naltrexone has in preliminary studies indicated an analgesic efficacy in FM with a low incidence of adverse effects. However, in this study, the analgesic efficacy of LDN was not corroborated.

## Disclosures

The authors declare that the article is a transparent and accurate report of the research undertaken and that there are no conflicts of interest to disclose.

## Appendix A. Supplemental digital content

Supplemental digital content associated with this article can be found online at http://links.lww.com/PR9/A195.

## Supplementary Material
