# Supplementary material for: Low-dose naltrexone for treatment of pain in patients with fibromyalgia: a randomized, double-blind, placebo-controlled, crossover study
Source: Pain Rep. 2023 Jun 15;8(4):e1080. doi: 10.1097/PR9.0000000000001080 (PMC10789452; doi:10.1097/PR9.0000000000001080)
Supplement: Supplementary file 1 [file painreports-8-e1080-s001.pdf]

Supplemental Digital Content 1

Text describing study setting and source data

(three-digit number refers to the manuscript)

2.3.1 Study setting

The study used a randomized, double-blind, placebo-controlled, crossover design and the patients were randomized and allocated equally according to computer random table method. The pharmacy managed the randomization sequence generated by a web-based randomization site [1]. The sequence was generated using the second generator function, applying blocks of 10 and balanced permutations. Randomization was conducted in October 2015 with 140 patients in 14 blocks of 10 with naltrexone/placebo or placebo/naltrexone. Two randomization lists, one for MPC-G, identification numbers 1-70, and one for MPC-C, identification numbers 71-140, were made. Patients were included consecutively. The randomization lists were made in duplicate and stored at the pharmacy and at each center, respectively. The randomization lists were stored in secure and locked confines, only accessible by the P.I. (KB) at MPC-G and a sub-investigator (MUW) at MPC-C. At MPC-C only one allocation number was used, and the patient did not complete the study. In case of a medical emergency, the code could be individually unblinded. All patients carried a card regarding contact information. If required, the P.I. from MPC-G, or an officer on call at the manufacturing pharmacy, could be contacted by phone 24/7. Any patient was withdrawn from the study if experiencing significant side effects after intake of placebo/LDN. In case of pregnancy or if the patient wished to withdraw from the study, this was allowed without further explanation. Treatment was then discontinued, and any additional/ not used medication was handed over to the staff at MPC-G. The investigator/sub-investigator did ensure follow-up of possible side effects until termination. If a patient had to withdraw from the study to start treatment with opioids, LDN treatment was discontinued 24 hours prior to opioid treatment. New patients with new randomization numbers were allocated to replace drop-outs. Reasons for exclusion or withdrawal were registered in the e-CRF.

2.3.2 Source data

Study data were manually entered into an electronic Case Report Form (e-CRF). The e-CRFs were stored in a central database (TOPICA) managed by The Region of Southern Denmark. Encryption and backup servers secured data, and data were only accessible by authorized study personnel. The GCP units and the DMA were provided with direct access to the data. Hand-out questionnaires were

stored for each patient in physical CRFs. Data from these questionnaires were electronically transferred to e-CRF after each visit ensuring a low risk of data loss. After the last patient visit, data were stored in OPEN, a dedicated research registry in The Region of Southern Denmark. Both electronic and physical data are stored for five years after the end of the study.

Reference Supplemental digital content 1

[1] randomization.com

Supplemental Digital Content 2

Text describing miscellaneous questionnaires

(four-digit number refers to the manuscript)

2.8.2.3 Miscellaneous Questionnaires

The Supplemental File contains descriptions of Brief Pain Inventory – Short Form (BPI-SF) [5], Daily Sleep Interference Scale (DSIS) [5], Hospital and Anxiety Depression Scale (HADS) [6], PainDETECT [1], and Pain Catastrophizing Scale (PCS) [5].

Brief Pain Inventory – Short Form

The Brief Pain-Inventory-Short Form (BPI-SF) [5] is developed to examine the pain intensity and impact on physical functioning and quality of life. The questionnaire consists of nine questions and takes five min to complete. The patient is asked to mark on a body chart pain areas and to indicate the maximum and minimum intensity of the pain during the last 24 hours, the average intensity of pain, and the current pain. The patient indicates the current pain management and the treatment efficacy. The patient is asked to evaluate how the pain has had an impact on the patient's general activity, mood, ability to walk, normal job and housework, relationship to other people, sleep, and happiness. The response type varies between the items from yes and no to a 0-10 point numeric scale and to 0-100 %. The scoring is calculated by adding the Pain Severity Scores in questions 3, 4, 5 and 6, and the Pain Interference Scores in questions 9a, 9b, 9c, 9d, 9e, 9f, and 9g. The pain severity scores are summed and divided by 4 (total score 0-10). The pain interference score is divided by 7 (total score 0-10) [5]. In the present study BPI-SF was used to train patients in pain scoring.

Daily Sleep Interference Scale

Daily Sleep Interference Scale (DSIS) characterizes the interference of sleep due to pain. The patients is asked to fill out DSIS once daily and is included as a part of the study diary. The scale is an 11-point numeric Likert scale (0 = the pain did **not** interrupt sleep, 10 = the pain did completely interrupt sleep) [4].

Hospital and Anxiety Depression Scale (HADS)

Hospital and Anxiety Depression Scale (HADS) evaluates signs of anxiety and depression based on 14 questions regarding the patients' emotional experiences during the preceding week. Seven

questions evaluate signs of anxiety (HADS-A), and seven questions evaluate signs of depression (HADS-D). Each item of the questionnaire is scored between 0-3. The maximum score is 21 points, and scores of ≥ 11 points indicate likely or definite signs of either anxiety or depression [6].

PainDETECT

The PainDETECT questionnaire (PD-Q) evaluates the presence of a neuropathic pain component. The questionnaire contains seven questions grading various neuropathic pain characteristics, three questions on the temporal pain pattern, and one question concerning the presence of radiating pains. A score ≤ 12 points indicates a neuropathic component to be very unlikely (< 15%). A score of 13-18 points indicates that a neuropathic pain component cannot unambiguously be rejected. A score ≥ 19 points indicate a neuropathic component to be very likely (> 90%) [1].

Pain Catastrophizing Scale

The Pain Catastrophizing Scale (PCS) characterizes emotions, reflecting on previous pain experiences. The PCS contains 13 items on thoughts and feelings associated with the pain experience. The patient indicates the degree to which they experienced the thoughts and feelings on a 5-point ordinal scale (0 = not at all, 1 = to a slight degree, 2 = to a moderate degree, 3 = to a great degree, 4 = all the time). The PCS yields a summed score for the 13-items (0-52 points) and three subscale scores assessing rumination, magnification, and helplessness [2,3].

References Supplemental digital content 2

[1] Freynhagen R, Baron R, Gockel U, Tolle T. painDETECT: a new screening questionnaire to identify neuropathic components in patients with back pain. Curr Med Res Opin 2006;22:1911-1920.

[2] Quartana PJ, Campbell CM, Edwards RR. Pain catastrophizing: a critical review. Expert Rev Neurother 2009;5:745-758.

[3] Sullivan MJ, Thorn B, Haythornthwaite JA, Keefe F, Martin M, Bradley LA, Lefebvre JC. Theoretical perspectives on the relation between catastrophizing and pain. Clin J Pain 2001;17:52-64.

[4] Vernon M, Brandenburg N, Alvir J, Griesing T, Revicki D. Reliability, validity, and responsiveness of the daily sleep interference scale among diabetic peripheral neuropathy and postherpetic neuralgia patients. J Pain Symptom Manage 2008;36;54-68.

[5] Williams D, Arnold L. Fibromyalgia Impact Questionnaire (FIQ), Brief Pain Inventory (BPI), Multidimensional Fatigue Inventory (MFI-20), Medical Outcomes Study (MOS) Sleep Scale, and Multiple Ability Self-Report Questionnaire. Arthritis Care Res (Hoboken) 2011;63:86-97.

[6] Zigmond A, Snaith R. The hospital anxiety and depression scale. Acta Psychiatr Scand 1983;67:361-370.

Supplemental Digital Content 3

Text describing Quantitative Somatosensory Testing

(two-digit and three-digit number refers to the manuscript)

2.9 Quantitative Somatosensory Testing

2.9.1 Heat-capsaicin Sensitization

Brush Secondary Allodynia

A test area of 3 x 3 cm² on the volar aspect of the right lower arm was delineated by a marker. A computer-controlled, calibrated contact thermode (2.5 x 2.5 cm²; baseline temperature 32ºC; ramp rate ± 1ºC;) was manually applied to the test area. The thermode was heated (45ºC, 300 s), inducing a superficial first-degree thermal injury. Immediately after removing the thermode capsaicin cream (0.075%) was applied in a uniform layer of 1-3 mm thickness in the test area. The stroking allodynia area was assessed outside the test area by a calibrated brush (SENSELab Brush no. 5, Somedic AB, Sösdala, Sweden; length/width/thickness 20/15/5 mm). The brush stimulation started in normal skin with strokes at a rate of 1 cm/s continues inwards towards the center of the test area. The patient, with closed eyes, was asked to immediately report if and when the brush-stroke changes from normal sensation to a pricking, stinging, or unpleasant sensation. The location of change was delineated with a marker on the skin. By stimulating along eight radial lines converging from the periphery towards the center of the test area, the borders of stroking allodynia were delineated. The allodynia areas were calculated from the octagons (Table 7). Differences in allodynia area between BA1 (Day 1) and BA2 (Day 36) and, OA1 (Day 21) and OA2 (Day 56), respectively, were calculated (Table 7).

Pin-Prick Secondary Hyperalgesia

Secondary hyperalgesia is an increased sensitivity to pain stimuli in normal skin outside an injury area [1]. Pin-prick secondary hyperalgesia areas (SHA) were assessed with a weighted-pin instrument (WPI; 128 mN [2.606 kPa]; MRC Systems Heidelberg, Germany) [4]. The pin-prick stimulation started in normal skin outside the test area and was applied with a stimulation rate of 0.3 – 0.4 Hz (downward movement 1.5 s; upward movement 1.0 s; horizontal movement 0.5 s) with a distance of

1 cm between each stimulus. The WPI was held perpendicularly to the skin during stimulation. The patient was asked, with closed eyes, to immediately report if and when the pin-prick changes from normal sensation to a pricking, stinging, or unpleasant sensation. The location of change was delineated with a marker on the skin. By stimulating along eight radial lines converging from the periphery towards the center of the test area, the borders of SHA are delineated. The SHA area (square centimeters) for hyperalgesia was calculated. The SHA assessments were performed immediately after the stroking allodynia test. Differences in SHA between BA1 (Day 1) and BA2 (Day 36) and, OA1 (Day 21) and OA2 (Day 56), respectively, were calculated (Table 7).

2.9.2 Pressure Pain Thresholds

Assessments of pressure pain thresholds (PPTs) were performed with a calibrated pressure algometer (Algometer, Type 2, Somedic AB, Sösdala, Sweden) [3]. The pressure algometer with a rubber-tipped probe (circular stimulation area 1.0 cm^2) was applied perpendicularly to the skin with an increasing pressure rate of 10-30 kPa/s. The cut-off pressure limit was set to 400 kPa. When the perception of pressure changed to pain, the patient verbally indicated the change, and the assessor immediately terminated the stimulus by withdrawing the pressure probe. Assessments of PPTs were performed at predefined tender points and control points (cf. specific timelines Table 1). Differences in mean in PPT between BA1 (Day 1) and BA2 (Day 36) and, OA1 (Day 21) and OA2 (Day 56), respectively, were calculated (Table 7).

The designated number and location of tender points examined by pressure algometry

1. *Right occipital region at the insertion of Mm. suboccipitale.*

The tender point is located by palpation of the insertion of the right rectus capitis posterior major muscle at the bony margin of the occipital bone.

2. *Right medial upper part of m. trapezius.*

The tender point is located by pinching the muscle belly of m. trapezius at the upper mid-margin. The algometer is placed perpendicularly to the skin between the fingers of the examiner.

3. *Right paraspinal thoracic region (at midscapular level).*

The examiner locates the midpoint of m. infraspinatus. An imaginary horizontal line is drawn. At the point crossing the midline of the spine the nearest processus spinosus is identified. Three cm laterally from the processus spinosus the algometer is placed perpendicularly to the skin (corresponds to m. rhomboideus major).

4. *Right second costochondral junction.*

The tender point is located by palpating the incisura jugularis manubrium sterni, finding the joining point of the second costa.. The algometer is placed perpendicularly on the joint line.

5. *Right lateral epicondyle of humerus (2 cm distal).*

The tender point is located by asking the subject to rest the right arm at a table with the elbow in 90º and the lower arm resting in the mid-supinated position (the thumb pointing upwards). Then the right elbow's lateral epicondyle is palpated, proceeding to 2 cm distal of the joint line. The origin of the extensor muscles is palpated with the examiner's first and third finger. The algometer is placed perpendicularly to the skin between the examiner's first and third finger.**6.**

6. *Right knee region, medial fat pad (proximal of the joint line).*

The subject lying in the prone position is asked to extend the right leg. The examiner then palpates the right flexed (20-25°) knee joint, finding the medial fat pad just proximal to the joint line. The algometer is placed perpendicularly to the designated point.

The designated number and location of control points examined by pressure algometry

7. *Right dorsal forearm (the distal third).*

The control point is located by asking the subject (with the arm in the supinated position) to do a volar flexion of the wrist, locating the proximal flexor crease. The examiner then indicates a point 5 cm proximally to the proximal flexor crease in the midline. The algometer is placed perpendicularly at this point.

8. *Right lunula of the thumbnail (assessed with the thumb placed on the table).*

The control point is located by asking the subject to place the right thumb on a table with the finger pad resting on the table. The algometer is placed directly and perpendicularly on top of the fingernail.

9. *Right third metatarsal bone (dorsal aspect, at the center of the bone).*

The control point is located by anteriorly palpating the subject's third metatarsal bone. The algometer is placed perpendicularly on the middle of the dorsal surface of the bone.

2.9.3 Conditioned Pain Modulation Test

The CPM test was performed as a cold pressor test by asking the patient to submerge the left hand into recirculating cold water (10.0 ± 0.3°C, 60 s). The cold-water level was maintained 1-2 cm above the wrist, and the patients were told to spread the submerged fingers, allowing the cold water freely to circulate around the hand [2,5]. The patient´s hand or fingers were not allowed to touch the casing of the bath.

The test stimulus was by pressure algometry applied 10 cm cranial from the superior margin of the right patella at the rectus femoris muscle (with the patient in a sitting position with 90° knee-flexion). The CPM test started with a baseline assessment (PPT 1). The left hand was then submerged into the cold water for 60 s. Following the withdrawal of the hand from the cold water, an assessment (PPT 2) was performed (160-200 s) (cf. specific timelines Table 1). Differences in CPM between BA1 (Day 1) and BA2 (Day 36) and, OA1 (Day 21) and OA2 (Day 56), respectively, were calculated (Table 7).

The CPM efficiency was calculated as:

$$\text{CPM efficiency (\%)} = \frac{100*(\text{PPT 2-PPT1})}{\text{PPT1}}$$

The Conditioned Pain Modulation (CPM) test evaluates the efficiency of the descending inhibitory pathways and has been used as a quantitative measure of pain disinhibition in FM-Patients [5].

References Supplemental Digital content 3

[1] Brietzke AP, Antunes LC, Carvalho F, Elkifury J, Gasparin A, Sanches PRS, da Silva Junior DP, Dussán-Sarria JA, Souza A, da Silva Torres AL, Fregni F, Caumo W. Potency of descending pain modulatory system is linked with peripheral sensory dysfunction in fibromyalgia: An exploratory study. Medicine (Baltimore);2019;98:e13477.

[2] Jensen-Dahm C, Werner MU, Dahl, JB, Jensen TS, Ballegaard M, Hejl AM, Waldemar G. Quantitative sensory testing and pain tolerance in patients with mild to moderate Alzheimer disease compared to healthy control subjects. Pain 2014;155:1439-1445.

[3] Petersen K, Rowbotham M. A new human experimental pain model: the heat/capsaicin sensitization model. Neuroreport 1999;10:1511-1516.

[4] Ringsted T, Enghuus C, Petersen M, Werner MU. Demarcation of secondary hyperalgesia zones: Punctate stimulation pressure matters. J Neurosci Methods 2015;256:74-81.

[5] Yarnitsky D, Bouhassira D, Drewes A, Fillingim RB, Granot M, Hansson P, Landau R, Marchand S, Matre D, Nilsen KB, Stubhaug A, Treede RD, Wilder-Smith OHG. Recommendations on practice of conditioned pain modulation (CPM) testing. Eur J Pain 2015;19:805-806.

Supplemental Digital Content 4

Text describing blood sampling and analysis

(two-digit and three-digit number refers to the manuscript)

2.10 Blood sampling and analysis

2.10.2 Naltrexone and 6β-naltrexol plasma concentration measurements

Blood samples for the analyses of pharmacokinetics (PK), inflammatory factors (IF), and genetics (G) were taken. Blood samples are stored in the research biorepository (biobank) until the analyses are made. Blood samples for the analysis of IF/G factors will be stored for 5 years in the biobank for later proteome analysis in relation to chronic pain. After analysis, all excess material will be destroyed. Venous blood samples for PK research were collected on Days 1, 14, 21, 36, 49, and 56. On Days 1 and 36 (first day of treatment periods), samples were collected in the morning just prior to intake of medication and then subsequently after 15, 30, 45, and 60 min. On Days 14, 21, 49, and 56, medication was taken in the morning and samples collected during the clinical visit 1 to 3 hours later. The samples were collected in 4 mL lithium–heparin containing tubes. Directly after the collection, the samples were placed on ice. Plasma was obtained by centrifuging at 2,500 g for 10 min at 4ºC and thereafter the plasma was transferred into cryo-tubes (2 duplicates). The tubes were marked with randomization number, date, time, and treatment period. Samples were initially stored in a freezer at a maximum temperature of -20ºC and later at -80˚C for long-term storage. The temperature was noted accordingly to the procedure of the laboratory. The freezer was equipped with an alarm, and a laboratory technician was contacted via telephone in case of any problems. Analysis of the plasma samples was made by UPLC/MS-_MS analysis after protein precipitation and dilution of the sample. All pipetting was performed by a Hamilton Starlet robot in microtiter plates. 100 µL sample was mixed for 5 minutes with 200 µL internal standard solution containing 33 µg/L of d3-naltrexone and d4-6β-naltrexol in methanol/water with 57 mg/mL zinc sulfate -heptahydrate. After centrifugation of the plate for 20 min. at 3000 g 100 µL of the supernatant was transferred to a new microtiter plate and diluted with 100 mircol MilliQ water before being placed in the autosampler.

The analyses were conducted on a Waters Acquity ultra performance liquid chromatograph (UPLC) with Xevo TQ-S tandem mass spectrometer operated in electrospray positive ion mode. The chromatographic separation was achieved with a Phenomenex Kinetex biphenyl column, 2.6µ, 100Å, 150 x 3.0 mm. The mobile phases were A: 0.1% formic acid in water and B: Methanol. The initial

conditions were 40 % B for 1.00 min, then linear gradient to 100 % B at 2.80 min. The column was rinsed at 100% B from 2.80 – 4.00 min and finally re-equilibrated at 40 % B from 4.10 to 6.00 min. The liquid flow was 0.50 ml/min throughout and the injection volume was 20 µL.

The multiple reaction monitoring (MRM) transitions used are shown in Supplemental Digital Content Table 1.

Supplemental Digital Content Table 1.

**Compound**	**Precursor ion (m/z)**	**Product ion (m/z)**	**Cone (V)**	**Coll. (eV)**
Naltrexone quantifier	342.37	323.99	25	20
Naltrexone qualifier	342.37	269.71	25	20
D3 naltrexone	345.37	270.11	25	20
β6-naltrexol quantifier	344.28	307.98	25	20
β6-naltrexol qualifier	344.28	325.99	25	20
D4 β6-naltrexol	348.28	312.28	25	20

The analysis was calibrated by in-house prepared calibrators in plasma (0 – 100 µg/L) and internal control samples were prepared in the same way in four different levels from stock solutions prepared independently from the stock solutions for calibrators.

The quantitative method was validated regarding linearity, accuracy, intermediary precision and limit of quantitation (LOQ). The method was confirmed to be linear in the calibrated range from 0 – 100 µg/L by visual inspection. The accuracy was evaluated by calculating the recovery in the independently prepared control samples. Samples spiked with 2.5, 10.0 and 50.0 µg/L showed recovery of 113%, 103% and 102 % respectively. The intermediary precision was found to be 9.5% at 0.35 µg/L, 3.3% at 2.50 µg/L, 6.0% at 10.0 µg/L and 4.9% at 50.0 µg/L. LOQ was set at 0.015 µg/L where the signal to noise ratio was above 10.

Supplemental Digital Content 5

Text describing Adverse Events

(two-digit number refers to the manuscript)

2.11 Adverse Events

Definition

An Adverse Event (AE) is any untoward medical occurrence in a clinical study participant administered a pharmaceutical product and which does not necessarily have a causal relationship with this treatment. An AE can therefore be any unfavorable and unintended sign (including clinically significant abnormal values from relevant tests, such as clinical safety laboratory tests, ECGs, vital signs), symptom, or disease temporally associated with the use of an investigational Medical Product (IMP), regardless of whether it is considered related to the IMP.

A baseline symptom is any medical event in a clinical study participant that occurs after he signed the ICF up until the first administration of IMP.

A treatment emergent AE (TEAE) is any AE not present, prior to the initiation of IMP administration or any event already present that worsens in either intensity or frequency following exposure to the IMP.

Only TEAEs are collected in this study (i.e. events occurring between screening and the first IMP administration are regarded as baseline symptoms and should not be recorded in the AE log in the ECRF.

Serious Adverse Events (SAEs) is any AE that results in death, is life-threatening (this refers to an event in which the participant was at risk of death at the time of the event; it does not refer to an event that hypothetically might have caused death had it been more severe), requires inpatient hospitalization or prolongation of existing hospitalization, results in persistent or significant disability/incapacity, is a congenital anomaly/birth defect, is medically important (this refers to an event that may not be immediately life-threatening or result in death or hospitalization, but may jeopardize the participant or may require intervention to prevent any of the SAEs defined above).

Medical and scientific judgement should be exercised in deciding whether expedited reporting is appropriate in other situations, such as important medical events that may not be immediately life threatening or result in death or hospitalization but may jeopardize the participant or may require

intervention to prevent one of the other outcomes listed in the definition above. These should also usually be considered serious.

Monitoring

At every visit, patients are asked about experienced adverse events, and this is registered in the e-CRF. The diary will be gone through, and any adverse event is noted in the e-CRF. The adverse event is graded as mild, moderate, or severe. Adverse event reported spontaneously by patients will also be registered as well will adverse events observed by the staff.

Reporting

The sponsor is responsible for reporting any **Suspected Unexpected Serious Adverse Reaction** (SUSAR) according to the Medicines Act. The sponsor must make sure that every information about SUSARs that are deadly or life threatening is registered within 7 days. Within 8 days after the reporting, the sponsor must inform the Danish Health and Medicines Authorities (DMA) about all relevant information regarding sponsors and investigators following up on the reporting. All SUSARs must be reported to the DMA within 15 days after the sponsor has been made aware of the SUSAR happening. The sponsor is informed of **Suspected Adverse Events/ Suspected Adverse Reaction** (SAE/SAR) within 24 hours by the sub-investigator as soon as the sub-investigator knows about it. It is the sponsor’s responsibility to interpret if this serious adverse event (SAE) or serious adverse reaction (SAR) is unexpected according to the product résumé. All suspected, unexpected severe incidents are noted ad reported electronically to the DMA according to rules and regulations. All severe Adverse Events will be reported yearly, and every severe Adverse Events will, no matter the relation to the study, be reported to the Danish National Committee on Health Research Ethics.

Supplemental Digital Content 6

Text describing statistics

(two-digit and three-digit number refers to the manuscript)

2.12 Statistics

2.12.2 Sample Size Estimates

The calculation is based on FIQ-data from Younger and colleagues [2], where mean values (SD) in the LDN group of 28.8 (12.5)% and in the placebo group of 18.0 (14.6)% are given for pain reduction, which gives an ES of 0.61 (GPower*3.1.9.2, Kiel University, Germany).

The sample size estimates were based on a 1% chance of type I errors ($\alpha = 0.01$), 10% chance of type II errors ($\beta = 0.10$), non-parametric distribution (ARE-correction; paired analysis with Wilcoxon signed-rank test), and an estimated correlation coefficient (r) between the treatments of 0.3. The estimated sample size per center was 51, allowing complete analyses to be performed at each center.

Correcting for drop-outs, 70 FM-patients per center should be included with a total number of 140 FM-patients. The significance level at 0.01 was employed to avoid mass-significance (type I errors) due to examining the primary outcome at each center separately and from the combined data from the two centers. An independent statistician verified the sample size calculation prior to the submission of the protocol. Data processing started directly after the last visit of the last patient.

2.12.3 Statistical Data Processing

Our analyses focused on measuring the pharmacodynamic effects of LDN compared to placebo on a number of primary and secondary outcomes. To do this, we exploited our access to both baseline and outcome measures for all individuals under both active treatment and placebo. This allowed us to perform paired tests. First, baseline (BA) and outcome (OA) measures were transformed into measures of changes (Δ) for all variables (v) and for all individuals (i) under treatment with LDN or placebo:

$$\Delta v_i = v_i^{OA} - v_i^{BA}$$

To assess the pharmacodynamic effects of LDN, the differences between LDN treatment and placebo were examined. Further, for the primary outcomes the approach in Younger et al [2] was also used, namely a random effects model with fixed effects at the condition (placebo *vs.* LDN), period, and

treatment order (whether the patient received LDN in the first period or not) level. Before performing the statistical tests, the data distributions of the outcomes were assessed for normality. In general, the normality of the outcome measures was rejected. Therefore, a non-parametric approach to analyze outcomes was used, reporting medians and interquartile ranges (IQR). Formally testing the null hypotheses of no effects, paired Wilcoxon signed-rank tests were used to report the associated *P*-values and appropriate effect sizes (ESs). For completeness, the results of the corresponding parametric tests were reported in tabular form (mean [SD], *P*, 95% CI, ESs) (Table 6, 8).

Before performing the statistical tests, the data distributions of the outcomes were assessed for normality. Specifically, the distributions of the differences were considered:

$$\Delta \mathrm{v}_\mathrm{i}^{\mathrm{LDN-Placebo}} = \Delta \mathrm{v}_\mathrm{i}^{\mathrm{LDN}} - \Delta \mathrm{v}_\mathrm{i}^{\mathrm{Placebo}}$$
$$= \left(\mathrm{v}_\mathrm{i}^{\mathrm{LDN,OA}} - \mathrm{v}_\mathrm{i}^{\mathrm{LDN,BA}}\right) - \left(\mathrm{v}_\mathrm{i}^{\mathrm{Placebo,OA}} - \mathrm{v}_\mathrm{i}^{\mathrm{Placebo,BA}}\right)$$

A formal test and a visual inspection of normality for each outcome were performed. The Shapiro-Wilk test was used due to its superior power properties compared to, e.g., the Kolmogorov-Smirnov test [1]. For visual inspection of the data, Q-Q plots were used. Results from test of normality and Q-Q plots can be obtained from the corresponding author upon request.

References Supplemental digital content 6

[1] Razali N M, Yap B W. Power Comparisons of Shapiro-Wilk, Kolmogorov-Smirnov, Lilliefors and Anderson-Darling Tests. JOSMA 2011;1:21-33.

[2] Younger, J, Noor N, McCue R, Mackey S. Low-dose naltrexone for the treatment of fibromyalgia: findings of a small, randomized, double-blind, placebo-controlled, counterbalanced, crossover trial assessing daily pain levels. Arthritis Rheum 2013;65:529-538
